# Evidence mapping for decision making: feasibility versus accuracy – when to abandon high sensitivity in electronic searches

**DOI:** 10.3205/000236

**Published:** 2016-07-19

**Authors:** Barbara Buchberger, Laura Krabbe, Beate Lux, Jessica Tajana Mattivi

**Affiliations:** 1 University of Duisburg-Essen, Faculty of Economics and Business Administration, Institute for Health Care Management and Research, Essen, Germany

**Keywords:** Evidence mapping, review literature, search strategy, sensitivity, specificity

## Abstract

**Background:** Mapping the evidence is a relatively new methodological approach and may be helpful for the development of research questions and decisions about their relevance and priority. However, the amount of data available today leads to challenges for scientists sometimes being confronted with literature searches retrieving over 30,000 results for screening.

**Objectives:** We conducted an evidence mapping of the topic “diabetes and driving” to investigate its suitability for an evidence-based national clinical guideline. In addition, we compared a highly sensitive search with a highly specific one.

**Methods:** Based on a systematic review, our database searches were limited to publications from 2002 to present in English and German language.

**Results:** Due to the strongly focused topic and the limits, our sensitive search identified a manageable number of references including sufficient evidence to answer our research question. Using the specific search strategy, we achieved a reduction of citations by 25%, concurrently identifying 88% of relevant references.

**Conclusions:** Evidence mapping with the intention of gaining an overview of a research field does not require high level accuracy in contrary to systematic reviews. Keeping this distinction in mind, a mass of extraneous information will be avoided by using specific instead of highly sensitive search strategies.

## Zusammenfassung

**Hintergrund:** Evidence Mapping ist eine vergleichsweise neue methodische Herangehensweise, die für die Entwicklung von Forschungsfragen und Entscheidungen über deren Relevanz oder Priorität hilfreich sein kann. Allerdings stellt die Menge der heutzutage zur Verfügung stehenden Daten eine Herausforderung für Wissenschaftler dar, die zum Teil mit Ergebnissen von Datenbankrecherchen konfrontiert sind, die über 30.000 identifizierte Literaturstellen für ein erstes Screening umfassen.

**Ziele:** Zur Überprüfung der Eignung des Themas „Diabetes und Fahrerlaubnis“ als Fragestellung für eine evidenzbasierte nationale klinische Leitlinie wurde ein Evidence Mapping durchgeführt. Darüber hinaus wurde eine hoch sensitive mit einer hoch spezifischen Suchstrategie verglichen.

**Methoden:** Auf der Basis eines systematischen Reviews waren die Datenbankrecherchen auf den Zeitraum von 2002 bis heute begrenzt sowie auf deutsche und englische Publikationen.

**Ergebnisse:** Aufgrund der stark fokussierten Fragestellung und der Begrenzungen ergab die hoch sensitive Recherche eine handhabbare Menge von Literaturstellen, die ausreichend Evidenz enthielten, um die Fragestellung nach der Eignung des Themas für eine evidenzbasierte Leitlinie positiv beantworten zu können. Unter Anwendung der hoch spezifischen Suchstrategie wurde eine Reduktion der Literaturstellen um 25% erreicht, mit der 88% der relevanten Literaturstellen identifiziert werden konnten.

**Schlussfolgerung:** Um eine Übersicht über ein Forschungsgebiet zu erlangen, ist im Gegensatz zu systematischen Reviews, die zur Reduktion von Bias die Darstellung der vollständigen, identifizierbaren Evidenz enthalten müssen, keine Recherche von größter Genauigkeit erforderlich. Unter Berücksichtigung dieses methodischen Unterschieds kann unter Anwendung einer hoch spezifischen Recherche anstelle einer hoch sensitiven eine große Menge zu bearbeitender, aber überflüssiger Informationen vermieden werden.

## Background

The term evidence mapping describes a system targeting an overview of the extent, nature and characteristics of a research field [1], often covering a wide range of topics. In addition, it is defined as a “less systematic but nonetheless replicable methodology that allows an understanding of the distribution of evidence” [2] within a broad medicinal or public health area [3]. Further reasons for undertaking an evidence map include a need for the prioritisation of research questions [4], as well as the identification of evidence gaps, or possibilities for future research [5], [6], [7]. Drawing evidence maps of research fields may help policy-makers to take well-informed decisions and estimate the feasibility and potential costs of a systematic review [1], [8].

According to the Global Evidence Mapping Initiative GEM [9], the mapping methodology comprises three consecutive core tasks. At the beginning, the boundaries and context of the map have to be set by development of researchable questions. This can be done by expert consultations, preliminary literature searches, a mapping survey, an online survey, or a combination of these. Subsequently, the prioritisation of questions has to be undertaken. The second core task involves an evidence search and selection [9] as known by the systematic review methodology [10], [11]. Finally and for reporting, data concerning interventions and study design as well as detailed study characteristics have to be extracted [9]. Extensions of evidence maps may include scoping studies, additionally comprising narrative accounts of the literature identified [1], [8], [9]. Moreover, evidence gap analyses for planning of future research can be seen as another enlargement [9]. Systematic reviews may also be based on evidence maps, but in contrast to the target of covering a broad area they are usually focused on specific and well-defined research questions [3], [4], [8], [9]. Other important differences of systematic reviews compared to evidence maps and scoping studies are quality appraisal and synthesis techniques used for aggregating the results [1], [3], [9].

Developing and running literature searches is the methodological core of scientific overviews. The design of a search strategy can be a challenge [12], whereby finding the balance between sensitivity and specificity can be considered an art. With overly specific searches, the risk of missing relevant evidence increases, while overly sensitive ones “create too much workload by resulting in screening unnecessarily many hits” [13].

## Objectives

Our aim was to determine the value of creating a full evidence synthesis concerning the research topic “diabetes and driving” and to investigate its suitability for a national clinical guideline. We therefore conducted an evidence mapping and compared a highly sensitive systematic literature search with a highly specific one. The evidence mapping was intended to reveal the amount of existing literature in general, as well as the amount of high quality evidence, e.g. systematic reviews and controlled trials. Content analyses of the identified literature had not been commissioned.

## Methods

We developed a highly sensitive search strategy and systematic electronic searches were conducted in April 2014 using the databases Medline, Embase and The Cochrane Library. Without a clear intervention, we used a modified PICO scheme being reflected in our search strategy (see Attachment 1). Preliminary searches identified a systematic review published in 2006 [14], itself based on an exhaustive review published in 2004 [15]. Therefore, we decided to limit our searches from 2002 to present and to German and English language. Exclusion criteria were: inadequate study population, indication other than diabetes mellitus, research question other than diabetes and driving, and abstract publication only. Despite being quite unusual for evidence mapping, for the purpose of gaining a more detailed overview we also conducted an evidence classification according to the Oxford Centre of Evidence-Based Medicine [16], focusing the first three levels of evidence (LoE) including systematic reviews, controlled studies and cohort studies, but without the assessment of single quality criteria. Title and abstract screening and the evidence classification were undertaken by two independent reviewers, resolving disagreements by discussion, whereby a third reviewer broke a tie when necessary. All choices and decisions were only made at abstract-level. In order to answer the question of whether the amount of effort and time could be reduced, we conducted both, highly sensitive and highly specific searches. For both searches, we built blocks of terms for the categories diabetes, complications and driving (see Table 1 (Tab. 1) and Attachment 1).

Unusually for evidence mapping, we only had one research question that was even clearly built up, meaning that both search strategies were not very extensive: the highly sensitive one consisted of about 70 terms, including MeSH and EMTREEs, respectively, and using truncations, synonyms and related terms. The complete highly sensitive search string as well as the highly specific one can be found in Attachment 1.

## Results

Using the highly sensitive search strategy limited by language and time period, we identified 884 references (see Table 1 (Tab. 1)) according to our exclusion criteria, of which two citations corresponded with Level of Evidence (LoE) 1 persuing to Oxford Centre of Evidence-Based Medicine [16], four citations with LoE 2 and 26 with LoE 3. Following the highly specific string using the same interface, the total number of references was reduced by 25% to 656 entries, of which one was classified as LoE 1, three as LoE 2 and 24 as LoE 3. Dividing the combined highly specific search into single database searches showed one citation with LoE 1 being linked to Embase only and thus not identifiable by other Medline searches. The second citation with LoE 1 and one citation with LoE 2 were not linked with diabetes as a key word and a term concerning driving ability, respectively; therefore, they were not identifiable by the highly specific search string. Furthermore, we took the opportunity of a manageable number of retrievals to compare different database interfaces with the same specific search string. We didn’t use specific functions or filters for the comparison of search interfaces. The discrepancies observed in the overall number of references retrieved by searching Medline via different interfaces are striking: searching Medline via Elsevier resulted in twice as many references compared to Ovid and PubMed, whereas the number of relevant citations was almost the same. 

## Discussion

Compared to the results of a highly sensitive search strategy, the results of a highly specific search would have been sufficient for answering our research question concerning diabetes and driving in the context of an evidence mapping. Only four relevant publications with a higher LoE (1–3) could not be detected by the specific search which would have provided an adequate basis for the decision to initiate a full systematic evaluation.

The information overload nowadays evokes a need for strategies to cope with this. Richard Smith describes that a consistent proceeding according to the methods of evidence-based medicine can hardly be pursued because almost nobody has the time. He therefore proposes helpful machines as the ultimate solution [17], although even using electronic databases for searching the evidence may also aggravate problems with data flood rather than bringing relief [17].

For evidence mapping regarding prevention and treatment interventions for depression in young people, Callahan et al. [5] used a search strategy identifying 32,733 references. After the exclusion of 28,361 citations based on title and abstract screening, 4,372 potentially relevant references remained, which were ordered and screened as full-text versions. This is not a singular phenomenon, with Lakshman et al. [18] reporting 37,868 documents retrieved from an electronic literature database search, although it should be called into question facing time and budget constraints. 

It is certainly possible to screen up to 150 titles or abstracts in one hour, although it is hardly possible to do so effectively eight hours a day for five days a week due to dwindling concentration. Therefore, screening tens of thousands of references would take up to at least one month and cost a fulltime-job, if the screening process should be operated in a high quality manner by two independent reviewers. Ordering and procuring full-text publications would take additional time before thousands of full-text publications could be screened. Moreover, going back to the beginning of the process, building up and testing a complex search strategy also takes considerably more time than developing a specific search string. Therefore, the sense and purpose of broad literature searches should be well-conceived.

As in geography, where a small scale map is chosen to represent a target in detail and a high scale to provide an overview of a larger area, highly sensitive search strategies for evidence maps will produce high accuracy and precise estimates, albeit only at the end of a screening procedure starting with substantially larger numbers of retrievals compared to highly specific searches yielding less accuracy but also fewer irrelevant retrievals at the beginning.

To clarify the target, and in contrary to systematic reviews where it is absolutely necessary to identify the whole literature being relevant for avoidance of bias, the particular research questions and its consequences for the workload following must be thoroughly considered in evidence mapping. If it is intended to provide an overview, “a high scale” should be chosen because breadth rather than depth is the goal, like a small scale map of a continent compromising the handling of the surface and making an overview hardly possible. Parkhill et al. [19] tested a sensitive search strategy versus a specific one for evidence mapping regarding a clinical question, whereby they did not miss any reviews or trials that were of significance to six different research questions by using the highly specific search string. The number of references to be screened could be minimised from 2,599 to 1,818, reducing the workload even at the very beginning of the working process by 30%. 

This is the first empirical study concerning a research question from a public health area comparing a highly sensitive literature search and a highly specific one for evidence mapping. Our findings confirm the results of Parkhill et al. [19] regarding a clinical question by reducing the total number of citations by 25% and reaching an adequate level of references identified for drawing an evidence map. The differences between search results using divergent databases and interfaces are well known [20], [21]; for example, the default setting in PubMed will search by MeSH terms and text words simultaneously, whereas searches via Ovid identify references by MeSH terms only [21].

Based on our results identifying approximately the same number of relevant citations using different interfaces, we would prefer searches via Ovid or PubMed reducing the number of references to be screened by half compared to Elsevier and thus being clearly more efficient. However, this may not apply for certain topics, such as searching the literature for specific terms, e.g. aboriginal, which is currently undergoing evolving political and cultural terminology [21].

According to Katz et al. [3], the breadth and depth of pertinent evidence should be characterised by evidence mapping. Nonetheless, facing the enormous number of 28,361 irrelevant citations screened by Callahan et al. [5] we propose abandoning such targets in the case of evidence maps with the aim of creating an overview of a scientific area and thus a tolerable risk of reduced accuracy yet increased practicality and perfectly sufficient results for answering the research question. This is in accordance with the request of Sir Iain Chalmers and Paul Glasziou [22] to reduce avoidable waste in the production and reporting of research evidence to minimise “the time and resource requirement […]; burdening those preparing them with excessive requirements”. Saving time by the waiver of developing a highly sensitive search string, testing it, and screening meaningless references has both human and economic consequences and will help to remain up-to-date with the evidence [23]. It is not only the preparation of reviews that is time-consuming, but also the peer-review process for scientific publishing, which sometimes takes up to one year or even longer [24]. “As time allows” is the gentle recommendation of Booth et al. regarding searches for mapping reviews [25].

Our results are limited by a single and clearly focused research question with a very low total number of citations retrieved, being uncommon for evidence mapping. In addition, the evidence assessment took place at an abstract level lacking plenty of information. Nevertheless, our results in a public health question can serve as an appeal for thorough considerations about the sense, purpose, and consequences of the methods used for evidence mapping. Further research ascertaining our findings and recommendations concerning the combination of database and interface is necessary in a broad research question and should be at least expanded to the database Embase searched via different interfaces.

## Conclusions

Using highly specific search strategies instead of sensitive ones is fully adequate for evidence maps with the aim of covering mainly the breadth rather than depth of a research spectrum. In this case, a highly specific strategy should be given preference to save human and financial resources as well as avoiding the risk of a conclusion being out of date.

## Notes

### Competing interests

The authors declare that they have no competing interests.

### Ethical approval

Not required.

### Source of funding

None.

## Supplementary Material

Appendix: Search strategies

## Figures and Tables

**Table 1:**
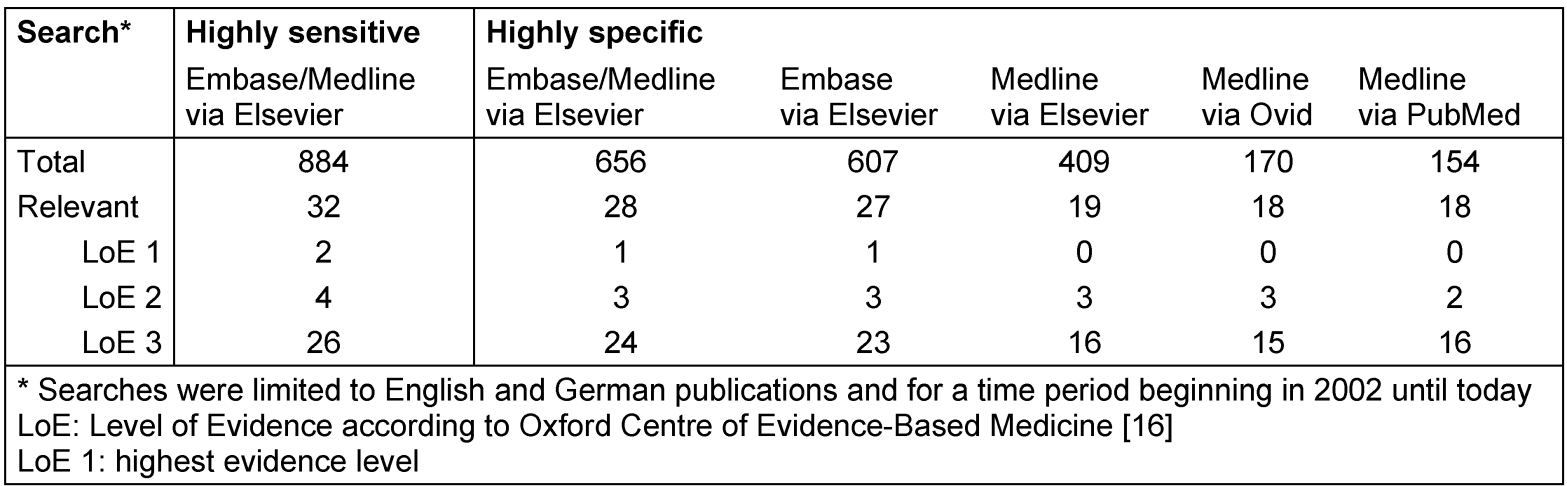
Retrieval of references, databases and search interfaces
