# Supplementary material for: Evidence mapping for decision making: feasibility versus accuracy – when to abandon high sensitivity in electronic searches
Source: Ger Med Sci. 2016 Jul 19;14:Doc09. doi: 10.3205/000236 (PMC4951635; doi:10.3205/000236)
Supplement: Appendix: Search strategies [file GMS-14-09-s-001.pdf]

Appendix: Search strategies

This appendix has been provided by the authors to give readers additional information about their work.

Sensitive search string
Databases: Embase/Medline, interface Elsevier

Blocks	Search terms
1 Diabetes	'diabetes mellitus' OR diabetes OR zuckerkrankheit OR
	EMTREE: *'diabetes mellitus'/syn*
2 Complications	hypoglyc*mia* OR hyperglyc*mia* OR 'diabetic ketoacidosis' OR 'coma diabeticum' OR 'diabetic retinopathy' OR 'diabetic neuropathy' OR hypoglyk*mie* OR hyperglyk*mie* OR hypoglyc*mie* OR hyperglyc*mie* OR 'diabetische ketoazidose' OR 'diabetische ketoacidose' OR 'diabetisches koma' OR bewusstseinsverlust OR 'diabetische retinopathie' OR sehverschlechterung* OR 'diabetische neuropathie' OR 'diabetic foot' OR 'diabetischer fuß' OR 'diabetisches fußsyndrom' OR 'diabetic foot syndrome' OR ('loss of consciousness' OR 'visual impairment' AND *'diabetes mellitus'/syn*) OR
	EMTREE: *'hypoglycemia'/syn* OR *'hyperglycemia'/syn* OR *'diabetic ketoacidosis'/syn* OR *'diabetic coma'/syn* OR *'diabetic retinopathy'/syn* OR (*'visual impairment'/syn* AND *'diabetes mellitus'/syn*) OR *'diabetic neuropathy'/syn OR 'diabetic foot'/syn*
3 Driving	car NEAR/1 driv* OR automobile NEAR/1 driv* OR 'motor vehicle' NEAR/1 driv* OR driving NEAR/1 licen?e* OR driver* NEAR/1 licen?e* OR 'automobile driver examination' OR 'driving ability' OR 'driving performance' OR driving NEAR/1 pattern* OR 'unsafe driving' OR 'driving impairment' OR 'driving safety' OR 'traffic safety' OR roadworthiness OR unroadworthy OR car NEAR/1 crash* OR traffic NEAR/1 collision* OR road NEAR/1 accident* OR traffic NEAR/1 accident* OR automobile NEAR/1 accident* OR 'motor vehicle' NEAR/1 accident* OR 'motor vehicle related injury' OR 'risk of injury' OR kraftfahrzeug* OR autofahrer OR autofahren OR führerschein OR fuehrerschein OR fahrerlaubnis OR führerscheinuntersuchung* OR fuehrerscheinuntersuchung* OR fahrt*chtig* OR fahrunt*chtig* OR verkehrst*chtig* OR verkehrssicherheit OR kollision* OR zusammensto* OR verkehrsunf*ll* OR verletzungsrisiko OR 'impaired driving' OR roadworthy OR 'road safety' OR car NEAR/1 accident* OR
	EMTREE: *'driving ability'/syn* OR *'car driving'/syn* OR *'driver licence'/syn* OR *'traffic safety'/syn* OR *'traffic accident'/syn*
(1 OR 2) AND 3 AND [2002-2014]/py AND ([english]/lim OR [german]/lim)	

Specific search string, interface Elsevier: (diabetes mellitus/ OR hypoglyc*mia* OR hyperglyc*mia* OR “coma diabeticum” OR “diabetic retinopathy” OR “diabetic neuropathy” OR “diabetic foot”), AND ((automobile NEAR/1 driv*) OR (driv* NEAR/1 licen?e*) OR “driving ability” OR (traffic NEAR/1 accident*)).

Attachment 1 to: Buchberger B, Krabbe L, Lux B, Mattivi JT. Evidence mapping for decision making: feasibility versus accuracy – when to abandon high sensitivity in electronic searches. GMS Ger Med Sci. 2016;14:Doc09.
DOI: 10.3205/000236
